# Optical-Spectrometry-Based Method for Immunosuppressant Medicine Level Detection in Aqueous Solutions

**DOI:** 10.3390/s18072001

**Published:** 2018-06-22

**Authors:** Marcin Marzejon, Monika Kosowska, Daria Majchrowicz, Barbara Bułło-Piontecka, Michał Wąsowicz, Małgorzata Jędrzejewska-Szczerska

**Affiliations:** 1 Department of Metrology and Optoelectronics, Faculty of Electronics, Telecommunications and Informatics, Gdańsk University of Technology, 11/12 Narutowicza Street, 80-233 Gdansk, Poland; marcin.marzejon.pg@gmail.com (M.M.); nika.kosowska@gmail.com (M.K.); 2 Department of Nephrology, Transplantology and Internal Diseases, Faculty of Medicine, Medical University of Gdańsk, 3a Marii Skłodowskiej-Curie Street, 80-210 Gdansk, Poland; bbullo@gumed.edu.pl; 3 Department of Morphological Sciences, Faculty of Veterinary Medicine, Warsaw University of Life Sciences, 159 Nowoursynowska Street, 02-776 Warszawa, Poland

**Keywords:** optical spectrophotometry, label-free detection, cyclosporine

## Abstract

In this paper, an investigation into detecting immunosuppressive medicine in aqueous solutions using a spectrometry-based technique is described. Using optical transmissive spectrometry, absorbance measurements in the spectra range from 250 nm to 1000 nm were carried out for different cyclosporine A (CsA) concentrations in aqueous solutions. The experiment was conducted for samples both with and without interferent substances—glucose and sodium chloride. Using a dedicated algorithm, the measured data was analyzed and a high correlation coefficient R^2^ = 0.8647 was achieved. The experiment showed that the described technique allowed for the detection of various CsA concentration levels in a selective, label-free and simple way. This method could be used in medicine, veterinary medicine and laboratory diagnostics.

## 1. Introduction

Cyclosporine A (CsA) is an inert, hydrophobic chemical compound. It is a cyclic polypeptide of 11 amino acids, whose molecular weight is 1203 [1]. It blocks humoral and cellular immune reactions, thus affecting the inflammatory reactions. CsA is used in the treatment of chronic inflammatory diseases in rheumatology, dermatology and ophthalmology [2,3,4]. However, long-term intravenous administration leads to serious side effects, including an increase of creatinine level, hypertension and the increased risk of cancer [5,6,7,8,9]. CsA contributes to water retention in the body resulting in the formation of edema and significant weight gains in people consuming this drug [10,11].

The use of cyclosporine in aquaculture animals, as well as its presence in water reservoirs, may contribute to an increase in mortality. Due to the legal regulations in force in the European Union, it is forbidden to use drugs that may contribute to the increase of the risk of disease and transmission of non-exotic diseases and the spread of exotic diseases in aquaculture animals [12,13]. The CsA presence in water will result in its accumulation in aquatic organisms. This can lead to various diseases due to the immunosuppressive action, causing e.g., nephrotoxicity, hepatotoxicity, among others. Consumption of aquaculture animals contaminated by CsA may lead to abnormalities in birds and mammals [14,15,16,17,18].

The main goal of this research is to detect CsA in water in general. It is possible to use the proposed method for measurements of any kind of aqueous solutions. It could be used for investigation of drinking water for animals, for water in reservoirs where the water animals live, for the waste water. This method allows for the detection of e.g., if the drinking water for animals contains CsA (this could be used for illegally caused gain of weight) or if the water in reservoirs is contaminated with this drug. In the future this method could also be used for testing drinking water for humans.

Out of several optical measurements methods, we chose the optical spectroscopy as the most promising [19]. In this paper, we present the optical spectrometry-based method for label-free detection of cyclosporine concentration in aqueous solutions.

## 2. Materials and Methods

### 2.1. Materials

A set of different aqueous solutions of cyclosporine was prepared in order to investigate if the proposed technique allows for the detection of CsA concentrations. Cyclaid^®^, produced by Apotex, and deionized water were used. Different weight percent concentrations [%] of CsA in deionized water were prepared according to Table 1.

### 2.2. Methods

A UV-9000 UV-VIS Double Scanning Spectrophotometer by Shanghai Metash Instruments Co. LTD was used for performing the measurements. In this spectrophotometer, a Deuterium Lamp and a Tungsten Halogen Lamp are used as light sources, a double beam optical system with 1200 lines/mm grating is implemented, and silicon photodiodes play the role of detectors. In this set-up, the light produced by the light source incidents on the diffraction grating and a spectrum is created. A part of it is directed to the beam-splitter. One of the beams passes thorough the sample and the other passes through the reference. The photodetectors measure the signal and the data is analyzed on a PC.

The spectrophotometer enables measurements in the spectra range from 190 nm to 1100 nm, with a bandwidth of 1.8 nm. The wavelength repeatability is about 0.2 nm, and the wavelength accuracy is equal to ±0.3 nm. Photometric accuracy is equal about ±0.3%T and photometric repeatability is equal to 0.2%T, where %T is percent transmittance. 10 series of measurements in the spectra range of 250–1000 nm were carried out with the increment of 1 nm.

## 3. Results

To establish whether optical spectroscopy can be used for detecting CsA in an aqueous solution, we performed a series of measurements for various CsA concentrations in water. A dedicated algorithm for measured data analysis was developed. A relationship between the drug concentration levels and the value of measured absorbance was established. Furthermore, the metrological parameters and selectivity of the method were established. Results of the aforementioned steps are shown in the following subsections.

### 3.1. Aqueous CsA Solutions

**Figure 1:**
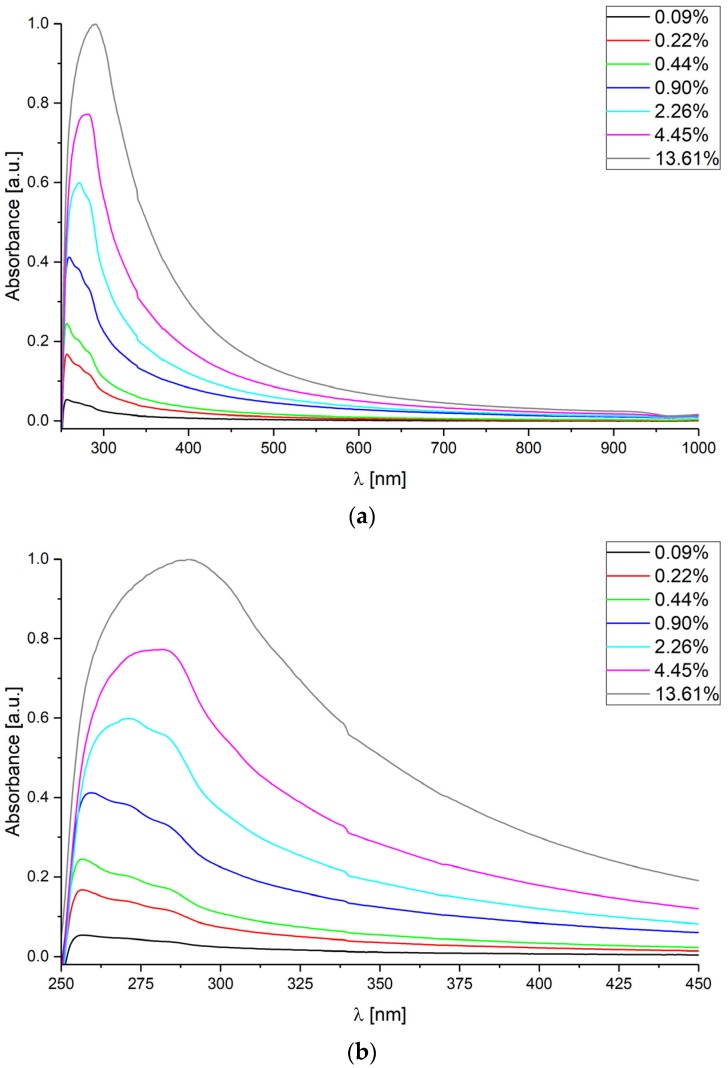
Absorbance spectra measured for various concentrations of cyclosporine A in deionized water in spectra range: (**a**) from 250 nm to 1000 nm; (**b**) from 250 nm to 450 nm.

In Figure 1, the acquired data for CsA concentrations varying from 0.09% to 13.61% in water is shown, using the measurement setup described in Section 2.2.

It is clearly visible that absorbance of CsA solutions increases, due to the increase of the CsA concentration level. The absorbance value changes rapidly in spectra range from 250 nm to 450 nm, which is shown in Figure 1b. The shapes of the measured absorbance curves are similar, regardless of CsA concentration, and the value of absorbance depends on CsA concentration. Furthermore, the wavelength of the observed peak *λ*_peak_, defined as the value of the wavelength for the highest value of absorbance, increases with the increase of CsA concentration level.

### 3.2. Selectivity of the Proposed Method

The next step was to investigate the effect of glucose C_6_H_12_O_6_ and sodium chloride NaCl presence in the measured samples on the measurements of CsA. We chose sodium chloride because it is the main ionic compound in water [20] and glucose because it impacts the aqueous animals in water ecosystems [21,22].

**Figure 2:**
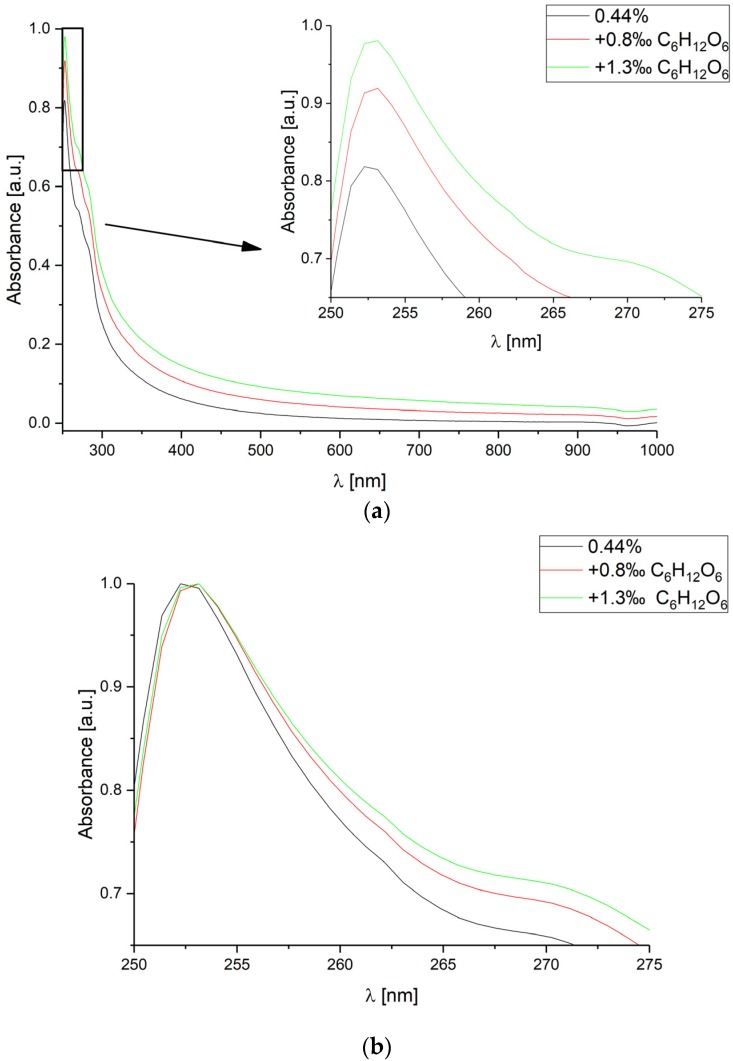
Absorbance spectra measured for various glucose C_6_H_12_O_6_ concentrations in deionized water, for constant cyclosporine A concentration level equal to 0.44%. The spectra range is: (**a**) from 250 nm to 1000 nm; (**b**) from 250 nm to 275 nm (each curve has been normalized separately).

Measurements for constant CsA concentration equal to 0.44%, and two glucose levels: 0.8‰ and 1.3‰ were performed. The measured absorbance spectra are shown in Figure 2.

For increasing glucose concentration level, the value of absorbance increases and the slope of the absorbance spectrum curve is smaller. Despite various glucose concentrations, the wavelength of the observed peak *λ*_peak_ is almost constant—slightly shifted by <1 nm.

**Figure 3:**
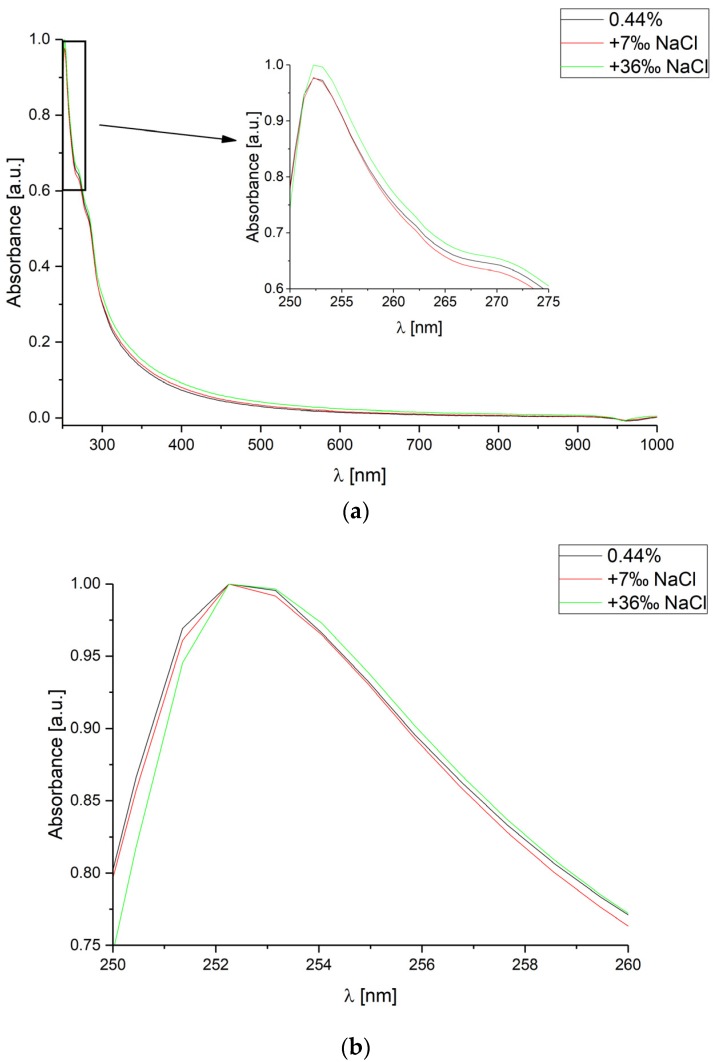
Absorbance spectra measured for various concentrations of sodium chloride (NaCl) in deionized water, for constant cyclosporine concentration level equal to 0.44%. The spectra range is: (**a**) from 250 nm to 1000 nm; (**b**) from 250 nm to 260 nm (each curve has been normalized separately).

Similarly, measurements for the same CsA concentration and two sodium chloride (NaCl) concentration levels: 7‰ and 36‰ were performed. The measured absorbance spectra are shown in Figure 3.

It can be observed that the investigated NaCl concentrations only slightly change measurement value of absorbance and could be omitted. Nevertheless, their influence might be significant for very low CsA concentrations. What is the most important, changes in the concentration level of NaCl do not affect the measured spectra shift.

As the next step, measurements for CsA concentration level equal to 0.44%, NaCl concentration level 36‰, and glucose concentration level 1.3‰ were performed. Similarly, the same measurements for CsA concentration equal to 4.45% were performed. The wavelengths of the observed peaks *λ*_peak_ for both CsA concentration levels are summarized in Table 2.

As it can observed, for different solutions the wavelength of the observed peak is almost constant—for selected solutions it is slightly shifted. This proves that the proposed method is insensitive to the influence of sodium chloride and glucose.

## 4. Discussion

### 4.1. Data Analysis Algorithm

**Figure 4:**
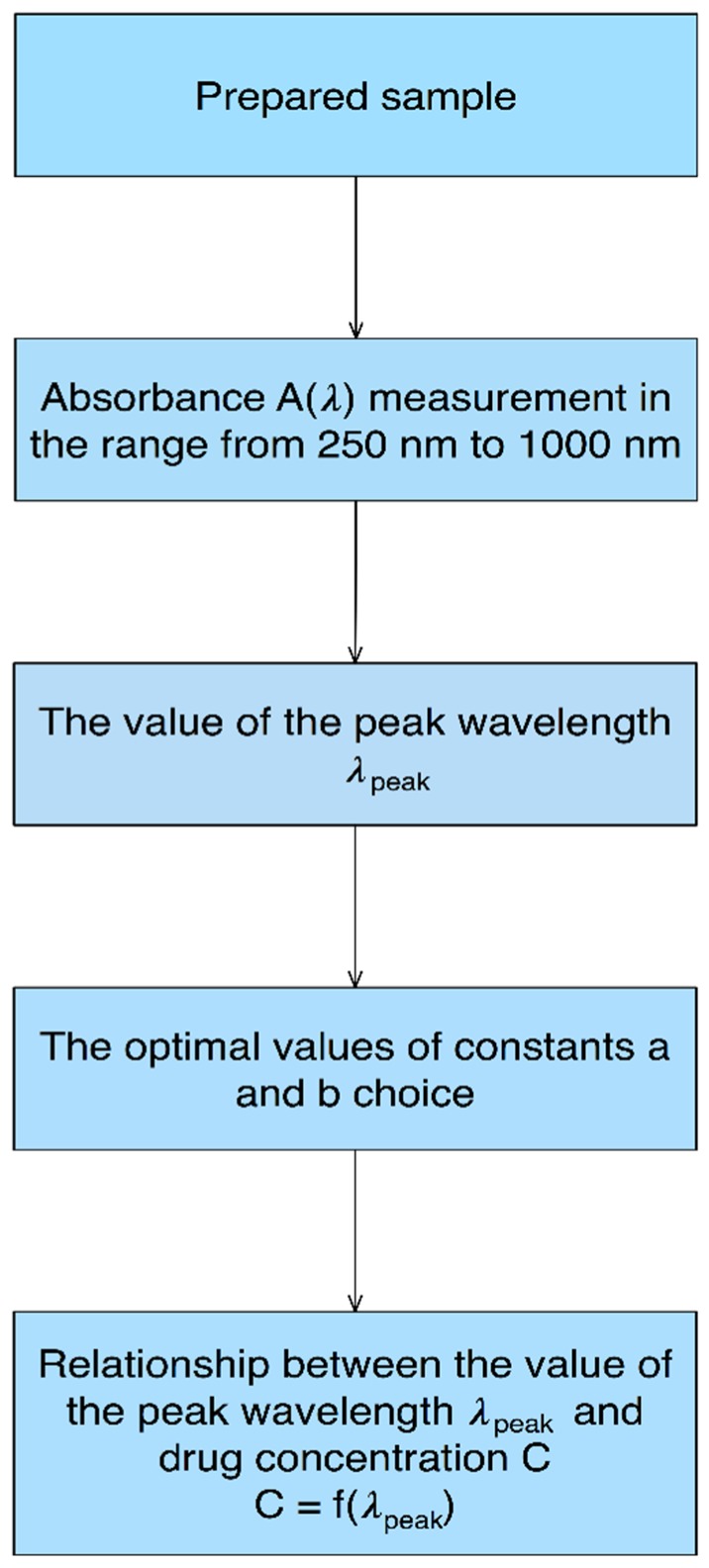
Data analysis algorithm schema. C is the drug concentration, *λ*_peak_ is the wavelength of the observed peak.

A dedicated data analysis algorithm was developed for measuring data analysis. It is illustrated in Figure 4.

For each CsA concentration, measurements of absorbance in the spectra range from 250 nm to 1000 nm were performed. Next, the wavelength of the observed peak *λ*_peak_ for various CsA concentrations was found. Those values were described by mathematical curves, using parameters a and b.

### 4.2. Relationship between the Value of the Peak Wavelength λ_peak_ and CsA Concentration

**Figure 5:**
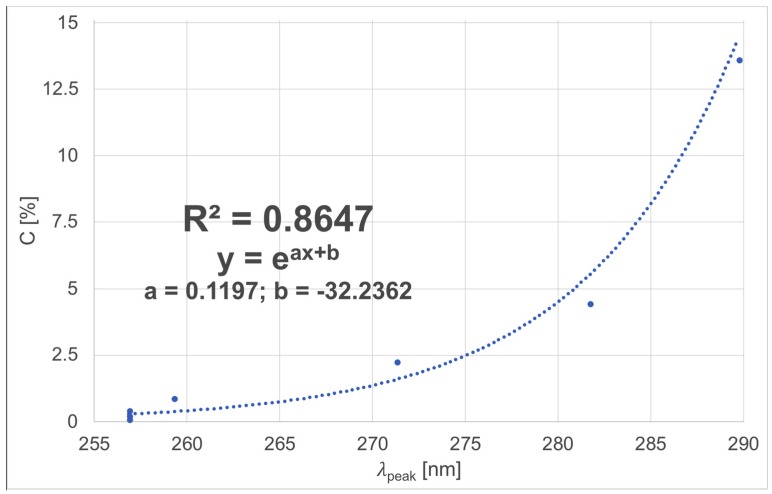
Relationship between the wavelength of the observed peak *λ*_peak_ and cyclosporine A concentration C for aqueous solutions.

The relationship between the wavelength of the observed peak *λ*_peak_ and CsA concentration C was calculated. It is shown in Figure 5.

We can observe that the relationship between the value of the peak wavelength *λ*_peak_ and CsA concentration C, shown in Figure 5 can be described by an exponential curve with parameters a and b. The obtained value of the coefficient of correlation R^2^ is equal to 0.8647. It proves that the selected mathematical model fits well with the measurements.

The obtained relationship enables us to calculate the value of the CsA concentration based on the wavelength of the observed peak *λ*_peak_.

## 5. Conclusions

In this paper, we presented the optical spectrometry-based method for detection of different concentrations of the immunosuppressive medicine—CsA—in aqueous solutions. The investigation showed that the correlation between CsA level and the value of absorbance can be found. Moreover, the proposed method is insensitive to the presence of investigated interferent substances, such as glucose and sodium chloride. The use of optical transmissive spectrometry assures many advantages, e.g., short measurement time, label-free measurements, simplicity. This technique could be successfully used in biosensors, e.g., for veterinary medicine, where CsA could be illegally administrated to farm animals in order to increase their weight.

## Figures and Tables

**Table 1:** Concentrations of CsA in prepared aqueous solutions.

Sample	Cyclaid Mass [mg]	Deionized Water Mass [g]	CsA [mg] in 4 g of Sample
13.61%	630.10	4	65.74
4.45%	186.37	4	19.44
2.26%	92.30	4	9.63
0.90%	36.39	4	4.02
0.44%	17.75	4	1.85
0.22%	17.75	8	0.92
0.09%	17.75	20	0.37

**Table 2:** The wavelengths of the observed peaks *λ*_peak_ for various concentrations of sodium chloride NaCl and glucose C_6_H_12_O_6_ in deionized water, for constant cyclosporine concentration levels equal to 0.44% and 4.45%.

Solution	Peak Wavelength *λ*_peak_ [nm]	
CsA Concentration	0.44%	4.45%
CsA in water	252.25	272.07
+0.8‰ of C_6_H_12_O_6_	253.15	272.07
+1.3‰ of C_6_H_12_O_6_	253.15	273.87
+7‰ of NaCl	252.25	272.07
+36‰ of NaCl	252.25	272.07
+1.3‰ of C_6_H_12_O_6_ + 36‰ of NaCl	252.25	272.07
